# Rolling dopant and strain in Y-doped BiFeO_3_ epitaxial thin films for photoelectrochemical water splitting

**DOI:** 10.1038/s41598-018-34010-9

**Published:** 2018-10-25

**Authors:** F. Haydous, N. D. Scarisoreanu, R. Birjega, V. Ion, T. Lippert, N. Dumitrescu, A. Moldovan, A. Andrei, V. S. Teodorescu, C. Ghica, R. Negrea, M. Dinescu

**Affiliations:** 1 Paul Scherrer Institut, Villigen, Switzerland; 2 National Institute for Laser, Plasma and Radiation Physics, 077125 Magurele, Romania; 3 National Institute of Material Physics, 077125 Magurele, Romania

## Abstract

We report significant photoelectrochemical activity of Y-doped BiFeO_3_ (Y-BFO) epitaxial thin films deposited on Nb:SrTiO_3_ substrates. The Y-BFO photoanodes exhibit a strong dependence of the photocurrent values on the thickness of the films, and implicitly on the induced epitaxial strain. The peculiar crystalline structure of the Y-BFO thin films and the structural changes after the PEC experiments have been revealed by high resolution X-ray diffraction and transmission electron microscopy investigations. The crystalline coherence breaking due to the small ionic radius Y-addition was analyzed using Willliamson-Hall approach on the 2*θ-ω* scans of the symmetric (00 *l*) reflections and confirmed by high resolution TEM (HR-TEM) analysis. In the thinnest sample the lateral coherence length (L_∥_) is preserved on larger nanoregions/nanodomains. For higher thickness values L_∥_ is decreasing while domains tilt angles (*α*_*tilt*_) is increasing. The photocurrent value obtained for the thinnest sample was as high as J_ph_ = 0.72 mA/cm^2^, at 1.4 V(vs. RHE). The potentiostatic scans of the Y-BFO photoanodes show the stability of photoresponse, irrespective of the film’s thickness. There is no clear cathodic photocurrent observation for the Y-BFO thin films confirming the n-type semiconductor behavior of the Y-BFO photoelectrodes.

## Introduction

Clean, environment-friendly energy production is one of the most important concerns nowadays, with major resources being allocated for developing new or improved ways to harvest energy from renewable sources. For photovoltaic or water splitting applications, metal oxides have proven limited performances due to the wide band-gap and the carrier transfer confinement at the junction. Moreover, there are important elements to consider when talking about the use of certain material for energy harvesting, excepting the absolute performances, such as the earth-abundant and production costs features for both the active material and the appropriate cocatalyst^1–3^. Small band-gap inorganic or hybrid perovskite materials are considered, from this point of view, the next step in clean energy production. The performances of organic-inorganic perovskite-based solar cells have a swift evolution, comparable to the commercially available thin films solar cells, as the latest reports are shown^4–6^. Progresses in durability and upwards scalability of production processes are, however, still underway.

Among inorganic perovskite materials, bismuth ferrite (BiFeO_3_, BFO) has a major advantage: the small value for direct band-gap (2.69–2.71 eV) compared with most ferroelectric materials^7–14^. Other interesting properties of this room-temperature multiferroic material, such as high remnant ferroelectric polarization (95 μC/cm^2^), Curie temperature (T_C_~1103 K) or ferromagnetism, have been intensively studied^15,16^. Reports on the photovoltaic properties of epitaxial BFO thin films deposited on DyScO_3_ substrate using a step-flow method for achieving layer-by-layer growth mode, pointing out the above band gap generated photovoltages, have shown the potential of this material in harvesting solar energy^7^. The high photovoltages are due to the spontaneous polarization in ferroelectric materials which led to high built-in potential in the volume, the charge separation and drift being improved, in contrast with metal oxides. There are reports on BFO nanoparticles induced water splitting for hydrogen generation, three times more effective than the present industrial standard, titania P25 Degussa^17^. As in the case of metal oxide nanostructures, the biggest challenge in this case is to obtain a high conversion efficiency (above 11% in stable conditions), with low-cost materials and without any back-up technologies for recollecting the nanostructures used in the process.

The photoelectrochemical (PEC) responses of pure or doped BFO have been reported for thin film-based photoanodes^12,18,19^. For polycrystalline BFO thin films, incident photon to current conversion efficiency (IPCE) of 7% at 530 nm (1.5 V) has been reported^20^. Epitaxial BFO thin films deposited by PLD on SrRuO_3_ buffered SrTiO_3_ (001) have been found to promote hydrogen and oxygen evolution due to a large switchable polarization which can induce positive or negative polarization charges on the film’s surface^21^. The cathodic shift of the photocurrent onset was noticed for thinner samples and negative polarization charges on the film. The authors suggested the unassisted water splitting behavior of BFO epitaxial thin films, due to the straddling of the band edges of epitaxial BFO films with the water redox potential. Recently, the PEC properties of epitaxial BFO thin films deposited by PLD with different crystallographic orientations have been reported by J. Song *et al*.^22^. They showed that the ferroelectric domain structure can induce a variation up to 8,000% in the photocurrent and 0.330 V shift in the onset potential, implying the use of domain engineering in ferroelectric materials for effective charge separation and collection, for efficient water splitting photoanodes.

However, high leakage, chemical stability and small photocurrent density (order of nA/cm^2^ up to tens of μA/cm^2^) issues of BFO material have led to the use of metallic doping elements as possible solution to these problems. Rare earth dopants such as Gd ^3+^, La^3+^, Dy^3+^, Sm^3+^ or Y^3+^ have been used for improving functional properties, including photovoltaic and photocatalytic properties, of both bulk and thin film BFO^23–27^. To narrow the band gap of BFO, doping with La, Gd or Y has been used for different kinds of nanostructures^24–27^. Mukherjee *et al*. reported a higher photocatalytic activity for Y-doped BFO nanoparticles than for BFO, and the possibility of tuning the electronic band structure of BFO by Y doping^24^. In spite of these arguments, reports on Y doped BiFeO3 epitaxial films and the associated photoelectrochemical properties are not present in literature, to our knowledge.

In a previous paper we have emphasized the concomitant influence of small ionic radius Y^3+^ doping and epitaxial strain on the dielectric behavior of BFO epitaxial thin films^28^. It was pointed out that the small ionic radius Y dopant had rolled the host BFO structure into an alternation of compressive and tensile nanostripe domain. The high in-plane dielectric permittivity values and low dielectric losses are due to this nanoscale complex structure associated with the presence of competing rhombohedral and orthorhombic ferroelectric phases. The effects of Y doping on the photoelectrochemical performances of BFO thin films are analyzed in this paper, as a function of epitaxial strain relaxation. The peculiar crystallographic nanostructure of Y-BFO films with the nanostripe regions rolling throughout the film’s thickness, as revealed in the previous paper, can induce the energy band bending towards the film-electrolyte interface due to high built-in potential and mobility towards external stimuli, e.g. applied electric field, and/or incident light^28^. The evolution of PEC measured photocurrents with the epitaxial strain relaxation, in contrast with the previous published papers on undoped BFO, is presented for the first time for Y-doped BFO thin films.

## Results and Discussion

The Pulsed Laser Deposition (PLD) method has been employed for the deposition of 3% Y-BFO (selected composition from Bi_1-x_Y_x_FeO_3_ x = 0.03) thin films with different thickness values, from 20 nm to 75 nm. The thickness of the Y-BFO thin films has been obtained from spectrometric ellipsometry fits, as well as from cross-section transmission electron microscopy. All other experimental details are presented in the Methods section. As a compulsory condition for performing stable PEC measurements in high pH value electrolyte, Nb:SrTiO_3_ (001) conductive single-crystals have been used as substrates. The Y-BFO thin films with thicknesses between 20 nm and 75 nm have been deposited on as-received Nb:SrTiO_3_ (STON) substrates, without any thermal or chemical treatment except the standard cleaning procedure. It is worth mentioning that if a step-flow growth method on vicinal substrates is used, for homoepitaxy, supplementary chemical etching and thermal treatments are needed. In order to avoid changes in the electrical properties of the substrate, any surface treatment has been avoided and no special miscut STON substrates have been used.

**Figure 1:**
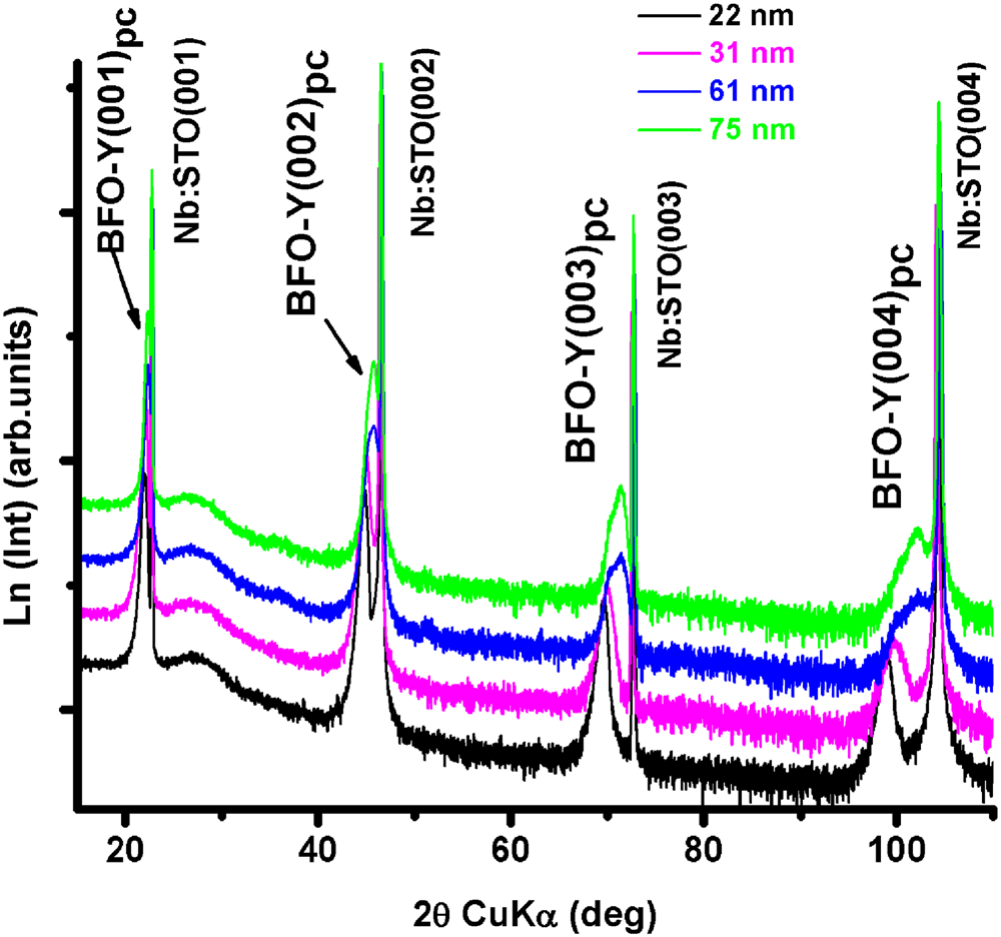
The 2*θ-ω* X-ray diffraction patterns of the Y-BFO thin films with different thickness values.

Figure 1 presents the standard 2*θ-ω* X-ray diffraction patterns of the Y-BFO thin films with different thickness values. All the films were crystallized in a pure perovskite phase without trace of any parasitic phase, as can be noticed from the (00l) reflections coming from of Y-BFO films and STON substrate. The calculated out-of-plane lattice constants (pseudocubic-pc unit cell considered) are obtained from these conventional symmetric (00l) reflections. The in-plane lattice constants are derived from off-axis diffraction scans at different tilt ψ angles for the four azimuthal angles.

The epitaxial nature of the Y-BFO thin films is demonstrated through XRD *φ-*scans, presented in the Supplementary Information (Fig. S1), where the presence of fourfold symmetry with “cube-on-cube” epitaxial growth can be noticed.

**Figure 2:**
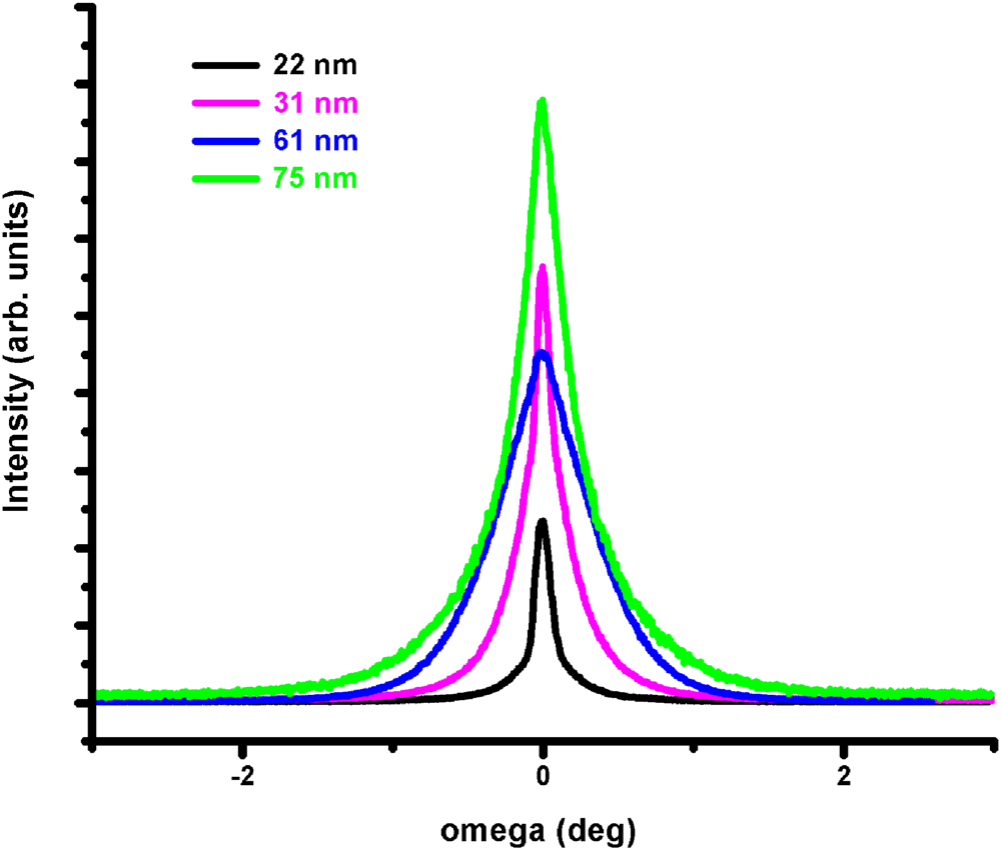
Symmetric x-ray rocking curves- ω-scans of the Y-BFO thin films with different thickness.

Symmetric x-ray rocking curves, *ω*-scans were used to disclose the mosaicity of the Y-BFO thin films. Figure 2 reveals the effect of the different thickness of the as-deposited Y-BFO films on the broadness of their (002) *ω* –scans.

The thinnest film, considered as fully strained, exhibited much smaller full width at half mximum (FWHM) values compared to the thicker films. These imply that due to strain, the mosaicity of the thin films is less affected by defects and dislocations and the coherence length is preserved on larger nanoregions/nanodomains. The Y-BFO lattice distortion towards out-of-plane direction is due to Y-addition and gradual relaxation of epitaxial strain with increasing the thickness, which favors extended defects to expand in the films. Superimposed rocking curves of the (002) peaks of Y-BFO and pure BFO thin films are presented in the Supplementary Information file (Fig. S2), for comparison purposes. As it was already observed in our previous work, the addition of the small ionic radius Y atoms to the BFO structure has a significant effect on the Y-BFO/STO thin films, especially on the “quality” of the epitaxial film expressed by its mosaic structure through crystalline lateral coherence lengths breaking^28^.

To gain additional structural information about the influence of small atomic radius Y dopant, X-ray diffraction peak broadening was analyzed as a function of films thickness via Williamson-Hall-type (W-H) approaches^28–31^. A traditional W-H treatment applied to the 2*θ-ω* scans of the symmetric (00 *l*) reflections allows the extractions of the vertical correlation length (L_ll_) and of the heterogeneous strain, (ε_┴_) along the c-axis, which causes the broadness of these peaks. Similar W-H plots performed on *ω*-scans around the same (00 *l*) reflections are used to find the lateral coherence length (L_ll_) and the mean mosaic domains tilt angles (*α*_*tilt*_). The calculated structural data are gathered in Table 1. An extended number of as-deposited Y-BFO films of different thickness measured through spectrometric ellipsometry fits were also investigated by XRD. The structural data including corresponding errors are presented in Table S1 (Supplementary Information).

**Table 1:** The structural data for Y-BFO thin films calculated using Williamson-Hall approach as a function of thickness.

Samples thickness (nm)	*a*_*out of plane*_ (Å)	*a*_*in plane*_ (Å)	*a*_out of plame_/*a*_in plane_	L_┴_ (nm)	ε_┴_ (%)	FWHM –*ω*(002) (deg)	L_II_ (nm)	α_tilt_ (deg)	Strain percent (%)
22	4.0473	3.910	1.035	21	0.15	0.145	36	0.493	3.65
31	4.0217	3.923	1.025	28	0.59	0.210	29	0.662	2.99
61	3.9834	3.954	1.007	60	0.63	1.125	14	1.543	2.01
75	3.9726	3.951	1.005	69	0.45	0.437	25	1.279	1.73

**Figure 3:**
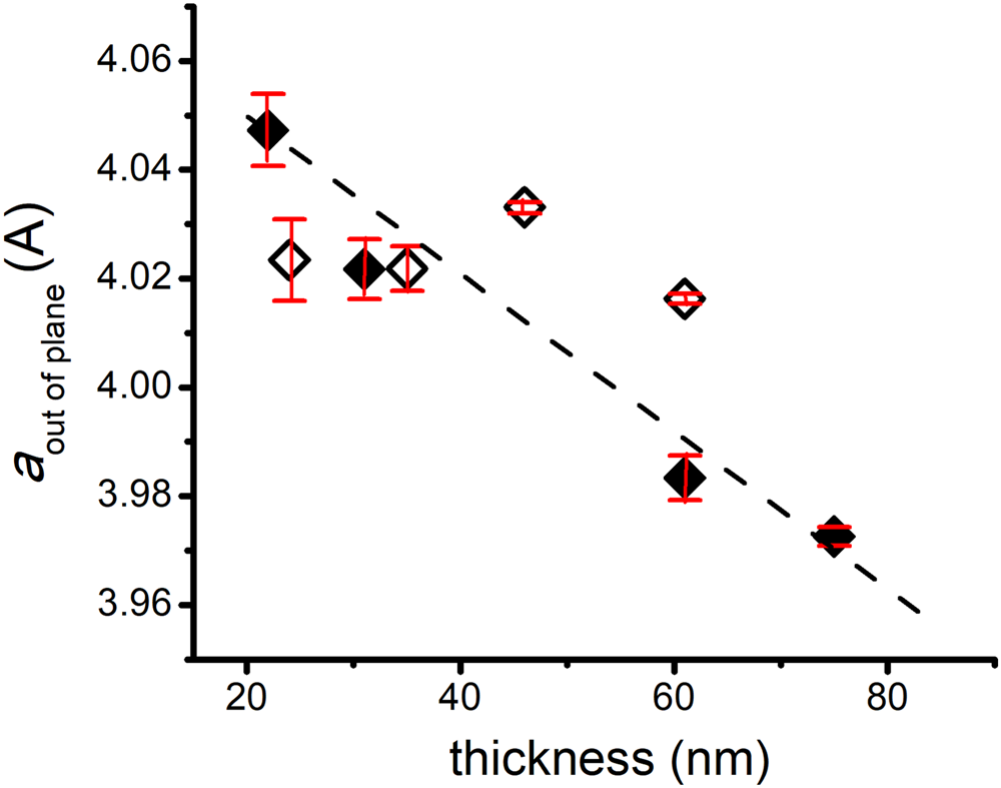
The *a*_*out of plane*_ values plotted for Y-BFO films with different thicknesses. Filled diamond symbols represent the values for the four tested samples while the empty diamond symbols stand for the supplementary deposited films with different thicknesses. The error bars represent the standard errors of the mean values.

The *a*_*out of plane*_ values for films with different thicknesses are plotted in Fig. 3. As expected, for the thinnest sample, the out-of-plane parameter has the highest value, *a*_*out of plane*_ = 4.0473 Å, due to fully substrate-induced strain state. All the values for the *a*_*out of plane*_ of Y-BFO films up to 75 nm in thickness, are higher compared to Bi_0.97_Y_0.03_FeO_3_ ceramics (about 3.94 Å). The in-plane *a*_*in-plane*_ value of 3.910 Å for the thinnest sample shows the fully strained state, being very close to the cubic STON substrate lattice parameter (3.905 Å). From the straight line fit of the plot in Fig. 3, a thickness of 145 nm corresponds to an out-of-plane parameter of 3.905 Å equal to the STON substrate lattice constant, thickness for which a Y-BFO film could be considered completely relaxed. This value was used to evaluate the strain percent defined as (*a* − *a*_*o*_)/*a*_*o*_ where *a*_*o*_ is the experimental lattice constant without strain, and *a* is the experimental lattice constant under compressive or tensile strain^32^. The strain percent values are presented in the last column of Table 1. As the thickness increases, the *a*_*in-plane*_ values follow the same trend, the relaxation of the strain taking place gradually. A similar behavior is observed in BFO films deposited on larger misfit substrates such as STO or STON, where strain relaxation with increasing thickness induces an increase of the in-plane lattice parameter accompanied by the decrease of the out-of plane lattice parameter^16,33,34^.

**Figure 4:**
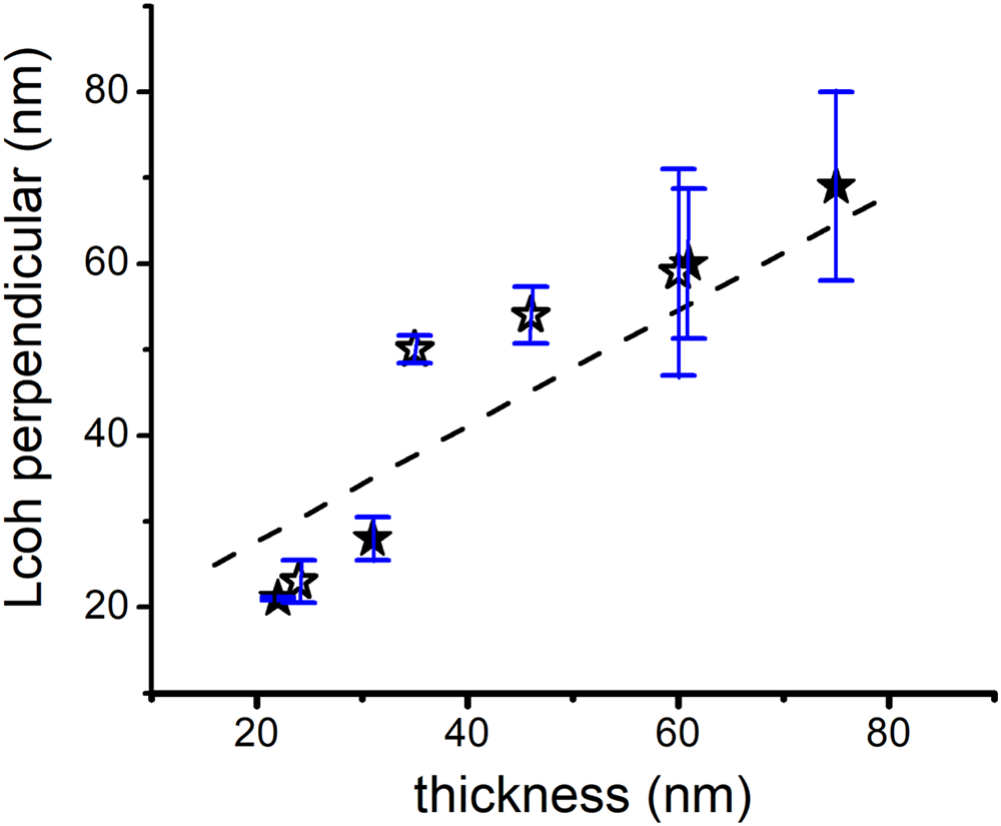
The out-of-plane (perpendicular) coherence lengths L_┴_ for Y-BFO thin films with different thicknesses. Filled star symbols represents the values for the four tested samples while the empty star symbols stand for the supplementary deposited films with different thicknesses. The errors bars are extracted from the standard deviation of intercept of the linear regression of W-H plots (*θ-ω* scans of the symmetric (00 *l*) reflections).

The out-of-plane, or perpendicular coherence lengths L_┴_, and the associated vertical “inhomogeneous strain” ε_┴_ values are caused by defects, strain or composition gradients in *c*-direction (out of film plane direction). The out-of-plane coherence length is comparable with the nominal film thickness, as Fig. 4 evidenced. A limitation due to defects densities in particular in the region near the film/substrate interface should, however, be considered.

The coherence length parallel to the substrate surface, *L*_∥_ and the tilt angle *α*_tilt_ values, included in Table 1, characterizing the mosaicity of films, depend on the film thickness. The parallel coherence length values are roughly decreasing with the film thickness, meaning much smaller nanodomains grown along the substrate for the thickest sample compared to the thin ones.

**Figure 5:**
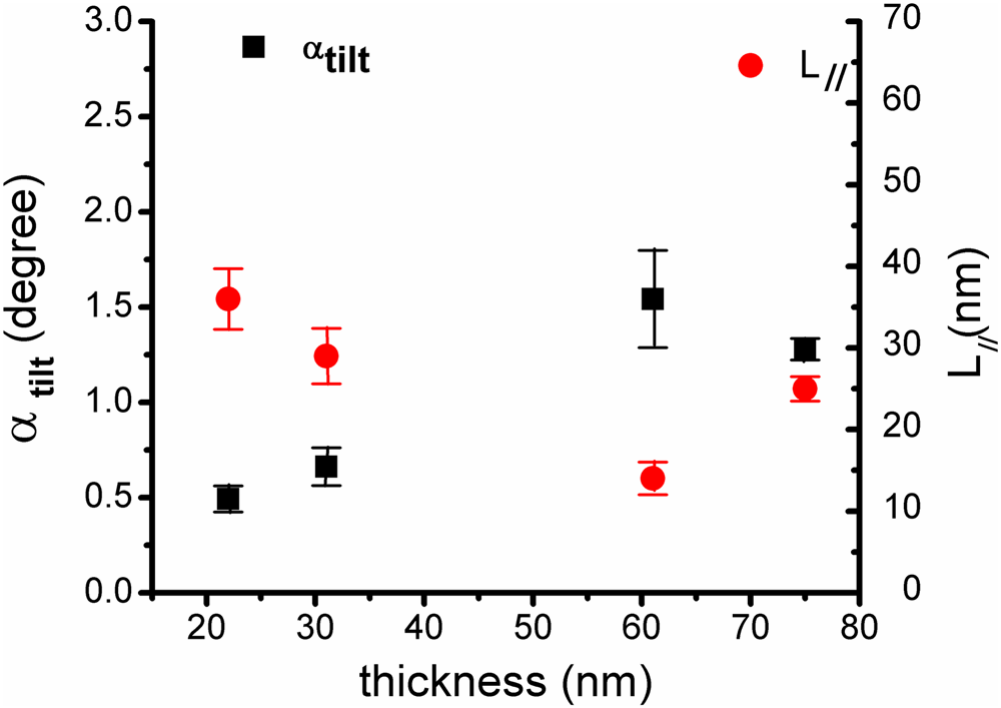
The coherence length parallel to the substrate surface *L*_∥_ and the tilt angle *α*_tilt_ values plotted as a function of Y-BFO films thickness. The errors bars are extracted from the standard deviation of intercept (for L_II_) and the standard deviation of the slope (for α_tilt_) from the linear regression of W-H plots (*ω*-scans around the same (00 *l*) reflections).

Following the same trend, the tilt angle is higher for high thickness values and sensible smaller for the 22 nm (and 24 nm) film(s) (Fig. 5).

**Figure 6:**
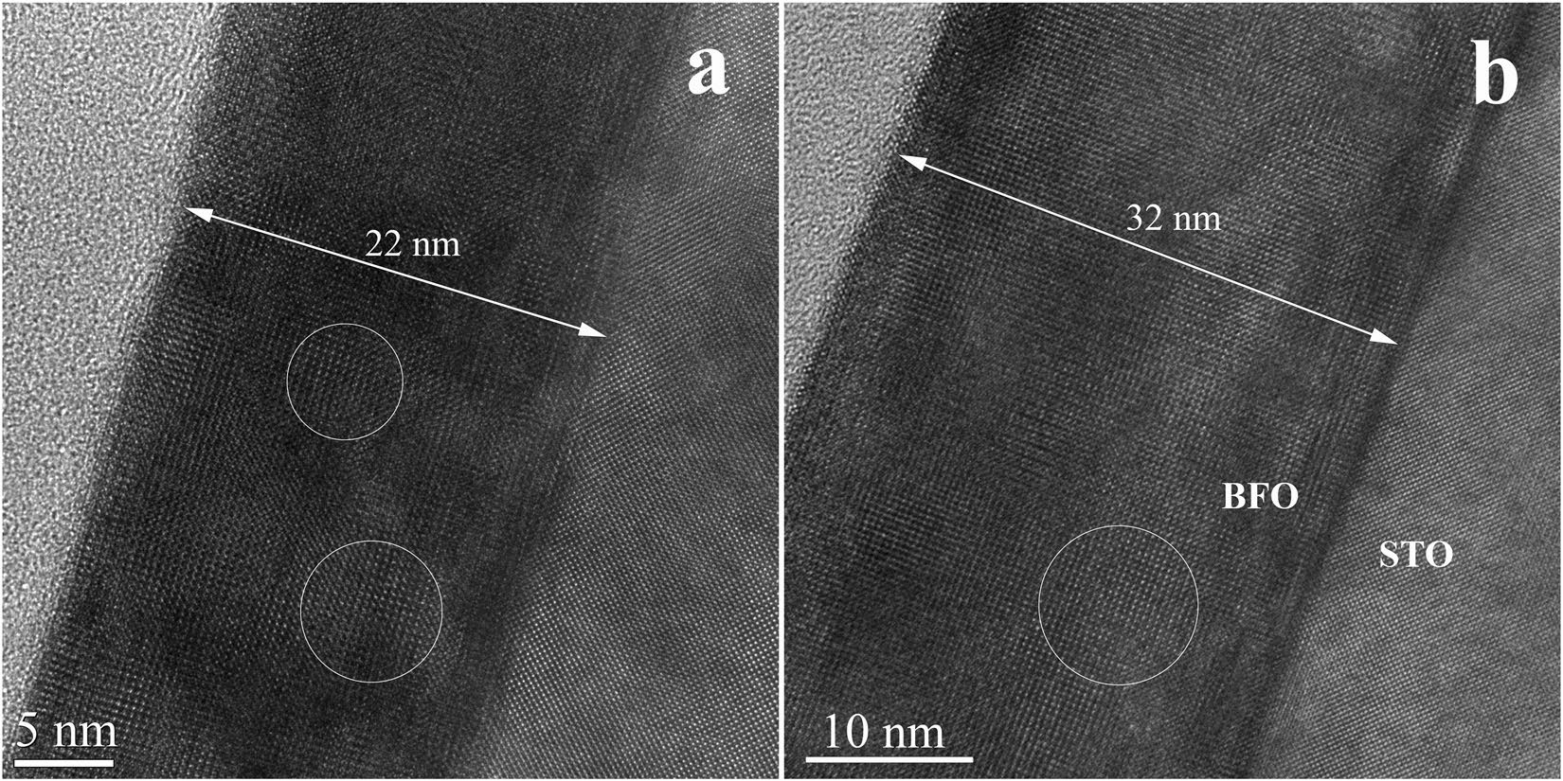
The HR-TEM cross-section images for theY-BFO samples with 22 nm (**a**) and 31 nm thickness (**b**). The coherent domains sizes are indicated by circles.

As stated above, the use of small miscut angle STON substrate without any surface treatments would lead to a Stranski- Krastanov type of growth for the Y-BFO films, the initial monolayers being followed by islands formation^35^. This type of growth mode has been revealed by TEM analysis also for samples in the present study, as shown in the Supplementary Information- Fig. S4. The HR-TEM cross-section images for the samples with 22 nm and 31 nm thickness, presented in Fig. 6, exhibit a defective film-substrate interfaces due to lattice mismatch between Y-BFO and STON substrate, for both samples. Moreover, differences in the lengths of the coherence domains, from 5–8 nm for 22 nm thick sample (Fig. 6a), to 7–11 nm in the case of 31 nm sample (Fig. 6b) are noticed.

**Figure 7:**
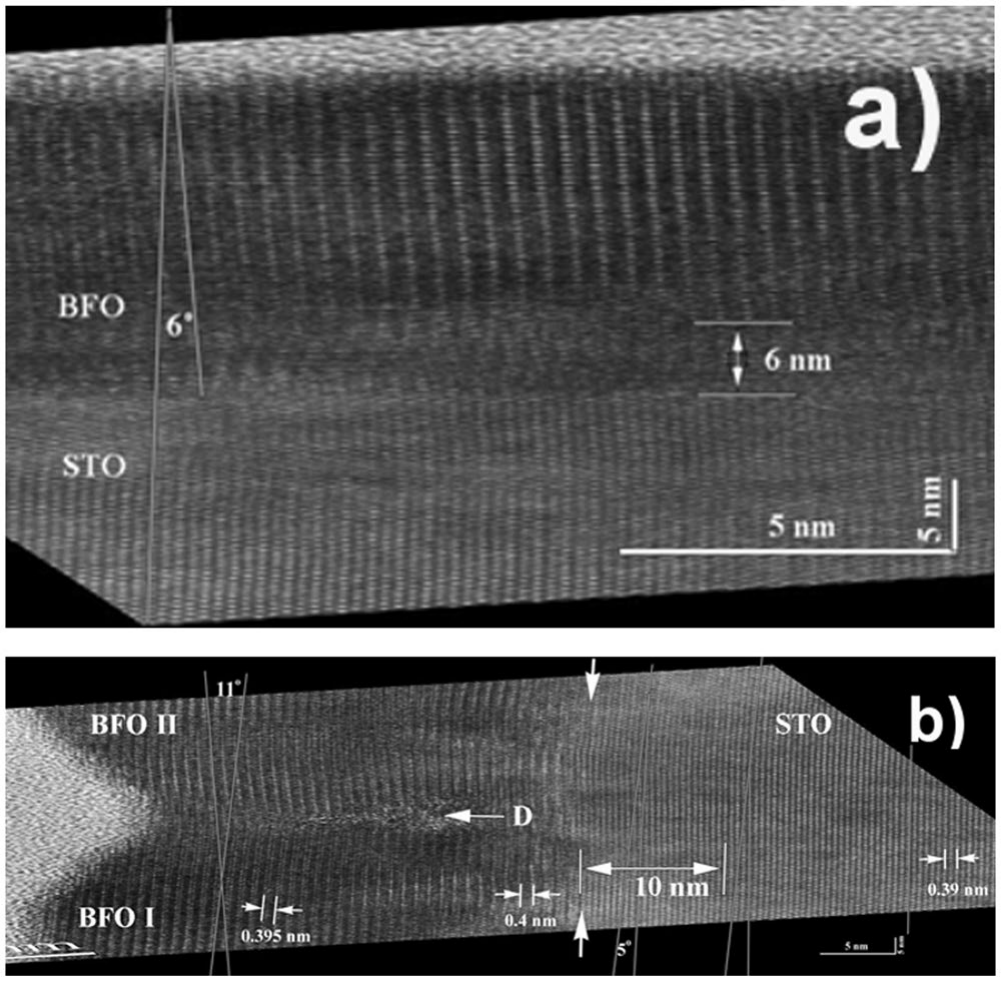
Glancing incidence analyzes performed on the HRTEM image of 22 nm thin Y-BFO sample along the Y-BFO film normal (N) (**a**) and along the Y-BFO/STON interface (P) (**b**). The inclination of the pseudo-cubic lattice plane of the epitaxial Y-BFO film respective to the STO substrate cubic plane, as well as the defected zones in Y-BFO and STO at the interface, are highlighted.

Glancing incidence analyzes were performed on the 22 nm thin Y-BFO sample by aligning the HR-TEM images along the [100] direction of the STON substrate and compressing the images five times along the Y-BFO film normal (N) or along the Y-BFO/STON interface (P) – Fig. 7a,b. These compressions correspond to a glancing incidence of 11.5 degrees, enhancing by a factor of five any misalignment of the lattice fringes parallel to the compressed direction.

Glancing incidence image along P compression- Fig. 7b, beside the lattice distorted region near the interface, shows defective region (D) at the interface between two neighboring crystalline coherent domains. Lattice planes parallel to the film surface and film-substrate interface are also visible. Moreover, the STON substrate lattice is disturbed (curved), especially under the defected region (D). The curvature of the STON lattice expands about 10 nm deep under the interface level. This curvature corresponds to the dilatation of the in-plane STON lattice and can be correlated with the STON tetragonal lattice which normally appears in the case of oxygen loss. The Y-BFO out-of-plane lattice distances near the STON interface are larger (0.4 nm) compared to the same distance (0.3950 nm) at more than 10 nm from the interface, showing the beginning of the relaxation process.

**Figure 8:**
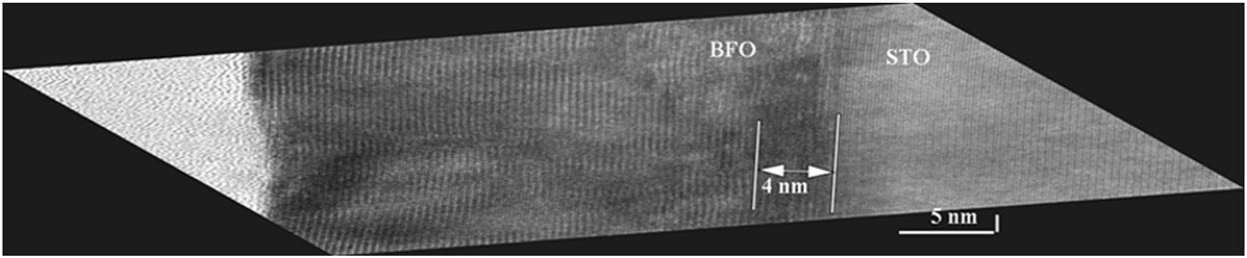
HR-TEM glancing incidence analyzes performed on the 31 nm thin Y-BFO sample along the Y-BFO/STON interface (P). The defected zone at the interface is located mainly in the BFO film in a depth of about 4 nm.

The defective region D at the interface between two neighboring crystalline coherent domains observed in the 22 nm Y-BFO sample, is not present in the thicker sample (31 nm)- Fig. 8.

**Figure 9:**
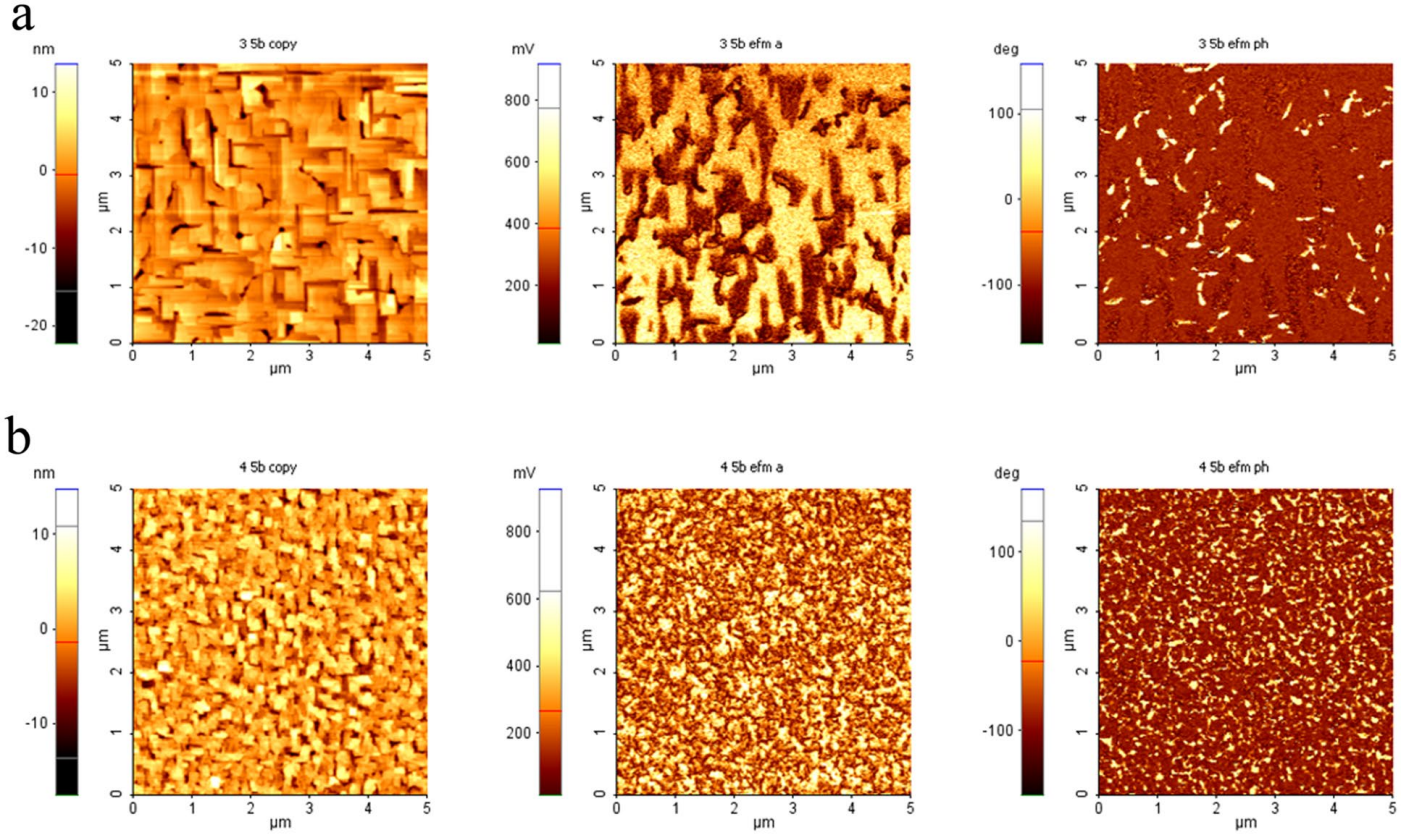
AFM topography (left) and PFM amplitude (center) and phase (right) images, recorded simultaneously on 5 µm × 5 µm areas of samples: pure BFO (**a**) and Y-BFO (**b**) thin films.

The crystalline coherence breaking due to Y-addition in BFO thin films is obvious also form the atomic force microscopy (AFM) and piezoforce microscopy (PFM) results (Fig. 9). On all the surfaces the PFM phase signal shows sharp variations between values separated by 180° (which proves the existence of domains with opposing orientations of the polarization vector). The phase offset of the AC signal applied to the tip was chosen in such a way to center at −90° and+90° the two distributions corresponding to these domains (dark and light regions, respectively). The fragmentation of the ferroelectric domains at the surface is clearly visible from the out-of-plane PFM response, the ratio between the number of the domains and their area being much higher for Y-BFO films (Fig. 9b), 31 nm thickness), in respect with BFO/STON sample (Fig. 9a), 33 nm thickness). The high domain contrast proves a strong lateral variation of the out-of-plane component of the polarization vector.

**Figure 10:**
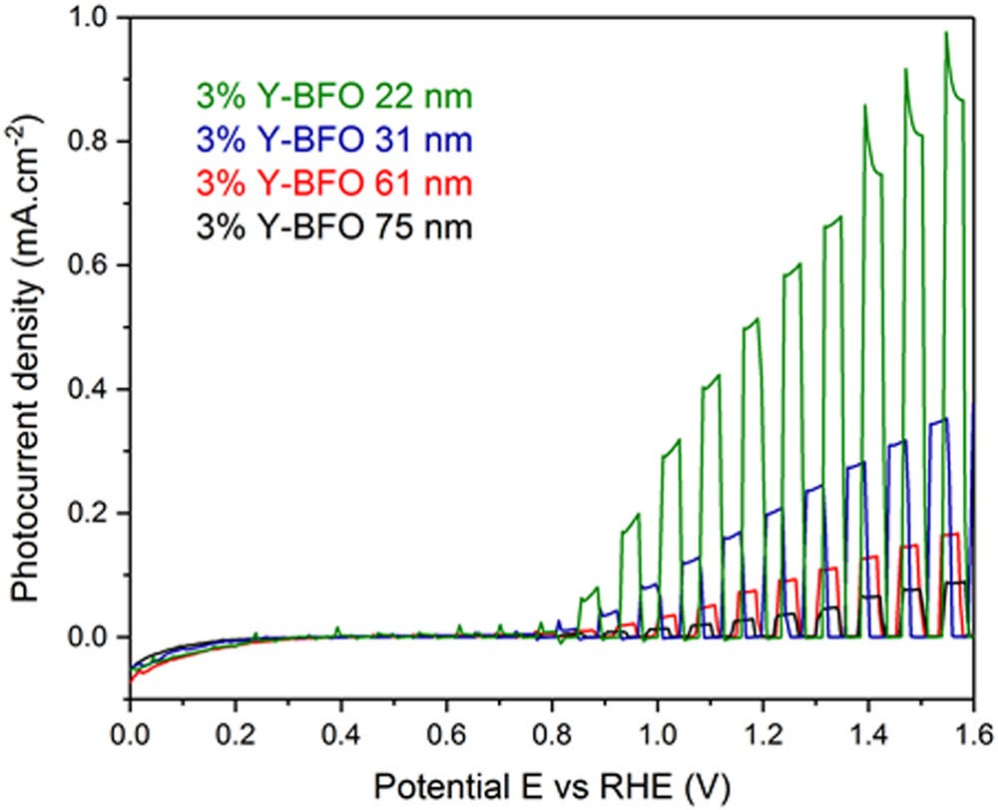
Potentiodynamic measurements performed for Y-BFO thin films up to 1.6 V vs RHE at a scan rate of 10 mV.sec^−1^.

Photoelectrochemical (PEC) measurements have been carried out to investigate the performance of the Y-BFO films in solar water splitting. The experiments were conducted in NaOH electrolyte using a three-electrode system, as detailed in the Methods section. Potentiodynamic measurements were first performed in the potential range of 0 to 1.6 V vs RHE at a scan rate of 10 mV.sec^−1^ under chopped light illumination using a 405 nm laser diode (5 mW output power, 3.1 eV photon energy). The light source was chosen so that any influence from the STON substrate (band gap 3.2 eV) can be neglected. Indeed, PEC experiments on bare STON substrates have been performed (see Fig. S5- Supplementary Information), to clarify any influence from the substrate. As can be observed in Fig. 10, a clear trend between the PEC performance and the thickness of Y-BFO films exists. The highest photocurrent, that is the difference between the light and dark currents, reached 0.72 mA.cm^−2^ at 1.4 V vs RHE for the thinnest film. The photocurrent values of the different films are reported in Table 2. For all the films, no clear cathodic photocurrent traces were observed, confirming the n-type semiconductor behavior of the Y-BFO photoanodes. The measured photocurrent values have a dependence on film thickness similar with the absorptivity coefficient *α*, shown in Fig. S6 (Supplementary Information). The dependence of the absorptivity on the films thickness is quite the opposite of the typical behavior of higher light absorption for thicker films exhibited by samples in which the optical constants are not changing with thickness. However, the difference of the light absorption at 405 nm irradiation wavelength between the thin and thick samples, seen from the absorptivity coefficient *α* versus thickness plots, is not the straightforward explanation for such high value of the photocurrent exhibited by the thinner samples. As mentioned above, the contribution of bare STON substrate to the photocurrents values is also negligible, as the potentiostatic scan at 1.4 V vs RHE under chopped light illumination shows. It can be seen from Fig. S5 that any influence of the substrate on the measured photoelectrochemical behavior can be excluded.

**Table 2:** The current density (J) and the absorbed photon-to-current conversion efficiencies (APCE) values at 1.4 VRHE as a function of Y-BFO films thickness.

Thickness (nm)	% Doping	J (mA.cm^−2^) at 1.4 V_RHE_	APCE
75	3%	0.06	0.001601
61	3%	0.13	0.003797
31	3%	0.3	0.011056
22	3%	0.72	0.031038

**Figure 11:**
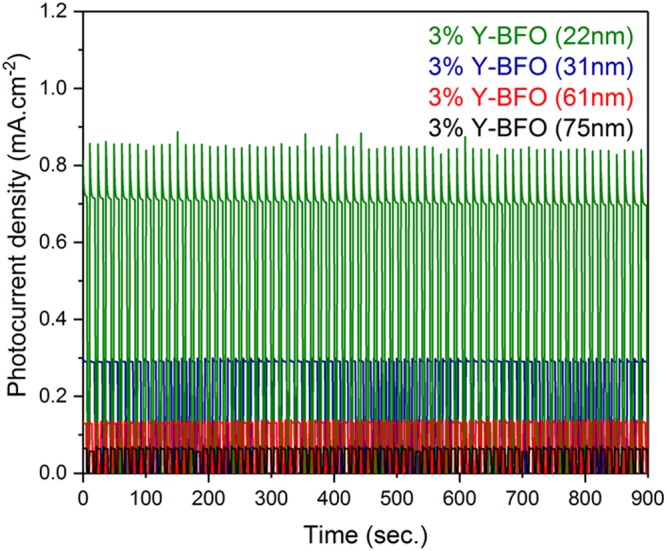
Potentiostatic measurements performed for the Y-BFO films at 1.4 V vs RHE for 900 s.

In order to study the stability of the Y-BFO photoanodes, the change in photocurrent at a fixed potential for 900 sec was studied. The resulting potentiostatic scans of the Y-BFO films at 1.4 V vs RHE are shown in Fig. 11. The Y-BFO photoanodes are stable, irrespective of the film’s thickness. Moreover, the structural changes of the films as a result of PEC measurements were investigated by XRD and presented in Fig. S3, Supplementary Information. All the PEC tested samples show structural stability, irrespective of the sample thickness.

As there are no reports, to our knowledge, on Y-BFO epitaxial film showing photoanodic behavior, the obtained photocurrent values are higher when compared to those reported for pure BFO thin films^21,22^. PEC properties of epitaxial BiFeO_3_ thin films under anodic bias, grown on SrRuO3/SrTiO_3_ substrates, have been reported by Ji *et al*.^21^. S. J. A. Moniz *et al*. have reported on the visible light driven water splitting by BFO thin films produced by dual-source low-pressure CVD, showing high IPCE values under 400 nm light irradiation^36^.

**Figure 12:**
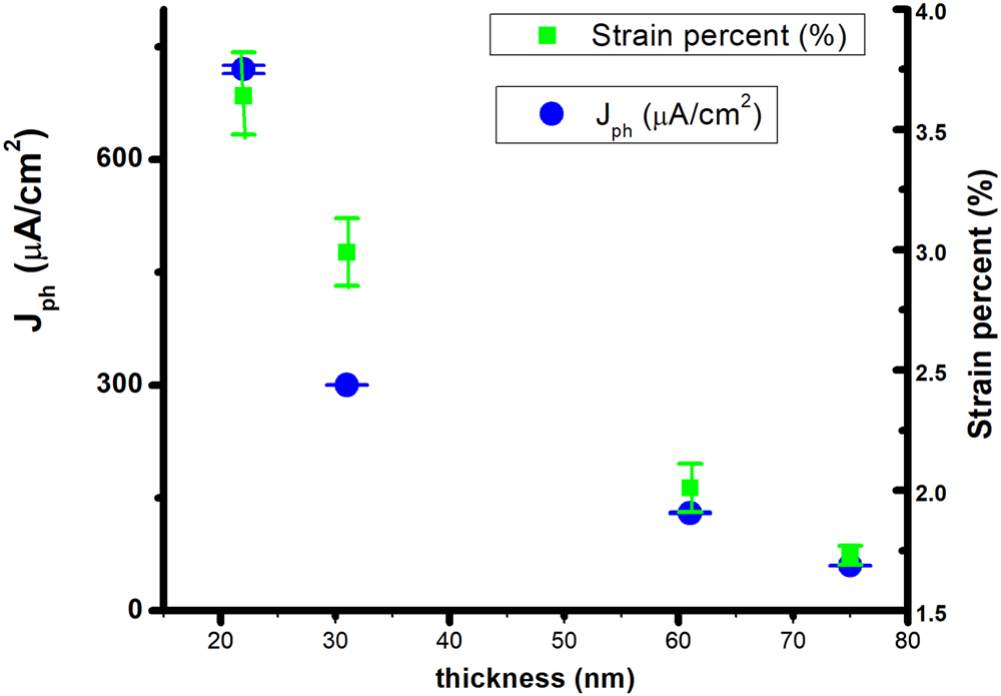
The photocurrent J_ph_ values versus Y-BFO films thickness and induced strain level. The errors bars represents the standard errors of the means values.

The recent paper of J. Song *et al*., presents the dependence of the PEC properties of BFO photoanodes on the crystalline orientation of the films and, consequently, on the different ferroelectric domain structure^22^. As the charge separation and collection driven by the electric field have been significantly improved by the ferroelectric switching in (111) BFO photoanodes, they show that the epitaxial BFO film’s thickness dependence of PEC activity is not a monotonical one. For epitaxial BFO films with thicknesses from 20 to 100 nm, the photocurrent density values increase with thickness, at 0 V vs. Ag/AgCl, up to 50 nm thickness, after which the photocurrent density decreases. The authors explained this type of photocurrent density dependence on thickness through the charge transfer model, where above an optimal thickness, the charge carrier recombination becomes critical, having a detrimental influence on the photocurrent^22^. This thickness is dependent on the carrier’s diffusion length of a material. In contrast to these findings, the Y-BFO films studied herein show a different trend for the photocurrent J_ph_ values versus thickness. As depicted in Fig. 12, J_ph_ follows closely the strain behavior exhibited by the Y-BFO samples as a function of thickness.

**Figure 13:**
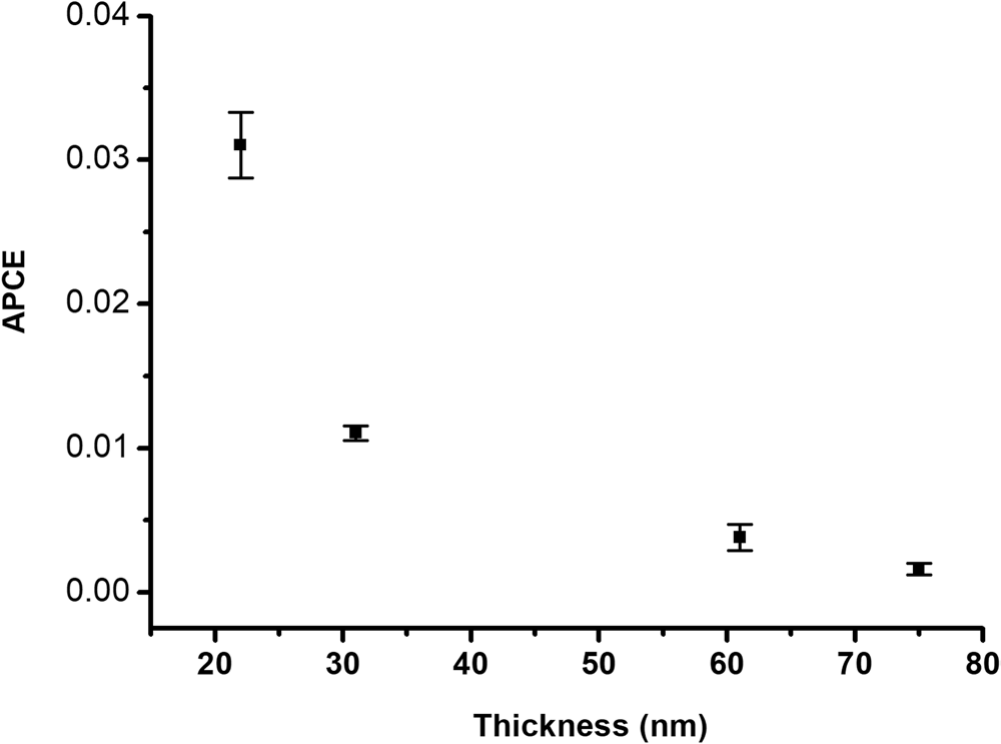
The absorbed photon-to-current conversion efficiencies (APCE) of the Y-BFO films.

However, the difference between the photogenerated currents for the 22 nm Y-BFO sample is more than two times higher if compared with 31 nm sample- Fig. 12. The explanation of photogenerated carriers’ recombination for such a high difference of photocurrent values, taking into account the relatively similar out-of-plane and parallel coherence lengths, as well as the mediated strain values, is not complete. The difference in the absorbance between the Y-BFO films of different thicknesses needs to be considered. For this reason, the absorbed photon-to-current conversion efficiencies (APCE) of the films were calculated and compared. As shown in Fig. 13, the APCE values showed the same trend as J_ph_ versus thickness, with the difference in the PEC performance being more evident between the 22 nm and 31 nm films. Recently, Y. Li *et al*. have presented the physical origins of a giant optical enhancement of the strain gradient in BFO thin films deposited on different substrates, by mapping of the spatio-temporal strain profile, strongly dependent on the film’s thickness^37^. They showed that, at the static strain in the films, the optical pumping adds supplementary enhancement of the strain gradient, thickness dependent. This enhancement of strain is explained by the appearance of piezoelectric effect due to the transient screening field mediated by excitons, being almost one order of magnitude larger for 20 nm BFO sample than for 35 nm one. Previously, the strain induced by the optical pumping was related to piezoelectric effects appearing due to the screening field created by the free carriers separated by the spontaneous polarization field. This model does not explain the thickness dependence reported by Y. Li *et al*.

## Conclusions

Epitaxial Y-doped BiFeO_3_ (Bi_0.97_Y_0.03_FeO_3_) films grown on Nb:SrTiO_3_(001) by pulsed laser deposition exhibit photoelectrochemical properties strongly dependent on the films thickness. PEC experiments revealed that the Y-BFO photoanodes are stable, irrespective the film’s thickness. The recorded photocurrent values follow the same trend with the film thickness as the induced strain, in contrast with previous results on BFO epitaxial thin films. The photocurrent value obtained for the thinnest sample was up to J_ph_ = 0.72 mA/cm^2^, at 1.4 V(vs. RHE). The lack of clear cathodic photocurrent traces for the Y-BFO thin films confirm the n-type semiconductor behaviour of the Y-BFO photoelectrodes.

## Methods

The films were grown by pulsed laser deposition on Nb: SrTiO_3_(001) (STON) single crystal substrates, from ceramic targets with composition Bi_0.957_Y_0.03_FeO_3_ (Y-BFO). The thickness of the films was set between 20–80 nm, in order to preserve the strained condition. The depositions were made under 13 Pa oxygen partial pressure, by using an ArF excimer laser (193 nm wavelength) with 5 Hz pulse frequency. The laser pulse energy was 22 mJ and the substrate temperature was 700 °C. In these conditions the growth rate was about 0.1 Å/s.

X-ray diffraction (XRD) at room temperature was performed with a PANalytical X’Pert MRD diffractometer, by using a line focused parallel monochromatic beam with CuKα1 radiation (0.1540598 nm) provided by a hybrid monochromator 2xGe(220) asymmetric.

The HR-TEM investigations were performed on a JEM ARM 200 F microscope.

Atomic force microscopy (AFM) and piezoforce microscopy (PFM) measurements were carried out on a commercial AFM (XE-100, Park Systems) in order to evidence the topography and the local ferroelectric domain orientation.

The optical properties of both substrate and films have been measured by ellipsometry technique (Woollam V-Vase system).

The PEC measurements were done using a three-electrode configuration with the Y-BFO thin films being the working electrodes, Pt and Ag/AgCl as the counter and reference electrodes.

The Y-BFO/STON samples have been electrically insulated on the back and sides, for avoiding any electrical short-circuit, with epoxy resin leaving only the thin films surface exposed to the electrolyte. Also, on the back of the STON substrate the electrical connection has been done using a conductive electrode and silver paste. The measurements were performed in 0.5 M NaOH (pH = 13) under chopped illumination from a 405 nm laser diode (130 mW.cm^−2^). The APCE values were calculated from the photocurrent density values using the following equation:

$$APCE=\frac{J(mA.c{m}^{-{\rm{2}}})\times {\rm{1239.8}}(V{\rm{.}}nm)}{{P}_{\lambda }(mW.c{m}^{-{\rm{2}}})\times \lambda (nm)\times A}$$

With the absorbance A calculated as shown below from the extinction coefficients (*k*) measured by spectroscopic ellipsometry:

$$A={\rm{1}}-{e}^{\frac{-{\rm{4}}\pi k{\rm{.}}t}{\lambda }}$$

## Electronic supplementary material

Supplementary Information
