# Supplementary material for: Rolling dopant and strain in Y-doped BiFeO3 epitaxial thin films for photoelectrochemical water splitting
Source: Sci Rep. 2018 Oct 25;8:15826. doi: 10.1038/s41598-018-34010-9 (PMC6202420; doi:10.1038/s41598-018-34010-9)
Supplement: Supplementary file 1 — Supplementary Information [file 41598_2018_34010_MOESM1_ESM.doc]

**Supplementary Information**

**Rolling dopant and strain in Y-doped BiFeO3 epitaxial thin films for photoelectrochemical water splitting**

F. Haydous^1^, N. D. Scarisoreanu^2*^, R. Birjega^2^, V. Ion^2^ , T. Lippert^1^ , N. Dumitrescu^2^, A. Moldovan^2^, A. Andrei^2^, V.S. Teodorescu^3^, C. Ghica^3^, R. Negrea^3^, M. Dinescu^2^

*^1^ Paul Scherrer Institut, Villigen, Switzerland*

*^2^* *National Institute for Laser, Plasma and Radiation Physics, 077125 Magurele, Romania*

*^3^National Institute of Material Physics, 077125, Magurele, Romania*

E-mail: [nicu.scarisoreanu@inflpr.ro](mailto:nicu.scarisoreanu@inflpr.ro)

**Contents:**

***Structural details for Y-BFO thin films.***

***Structural stability of Y-BFO thin films after PEC measurements.***

***Low magnification TEM images on Y-BFO films.***

***PEC measurement on bare STON substrate.***

***Optical properties of Y-BFO thin films and Nb:SrTiO3 used substrate.***

***Structural details for Y-BFO thin films***

| ***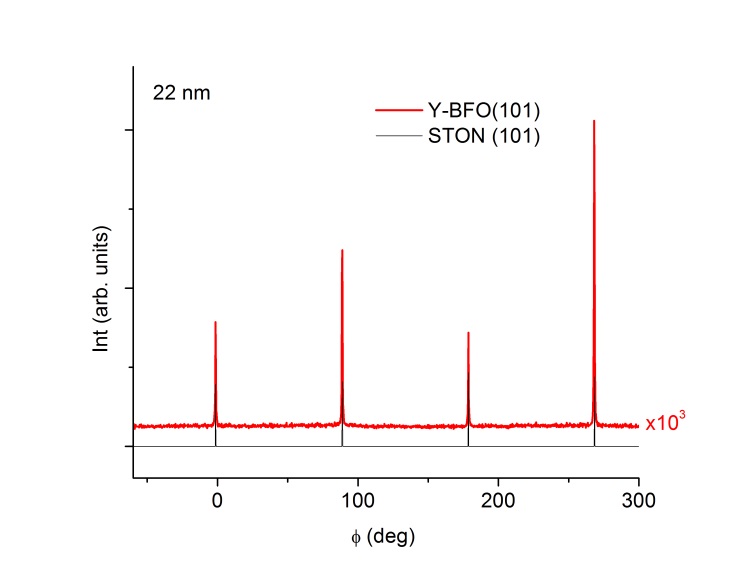*** | ***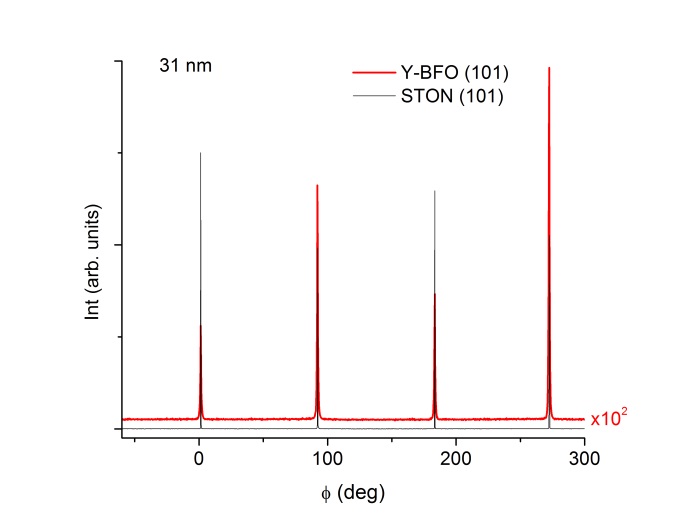*** |
| --- | --- |
| ***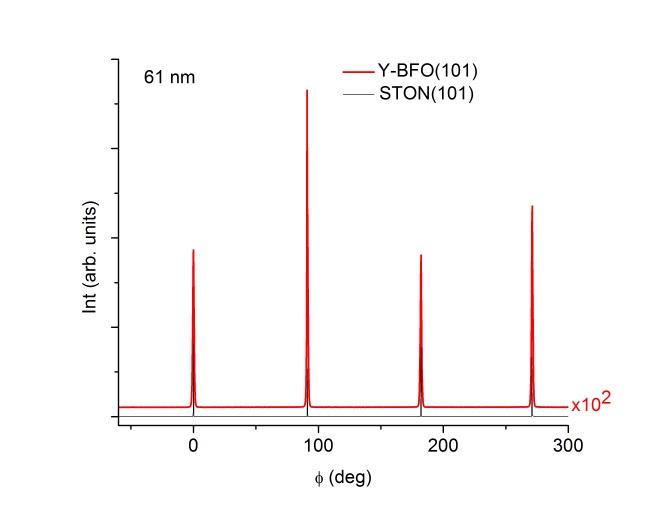*** | ***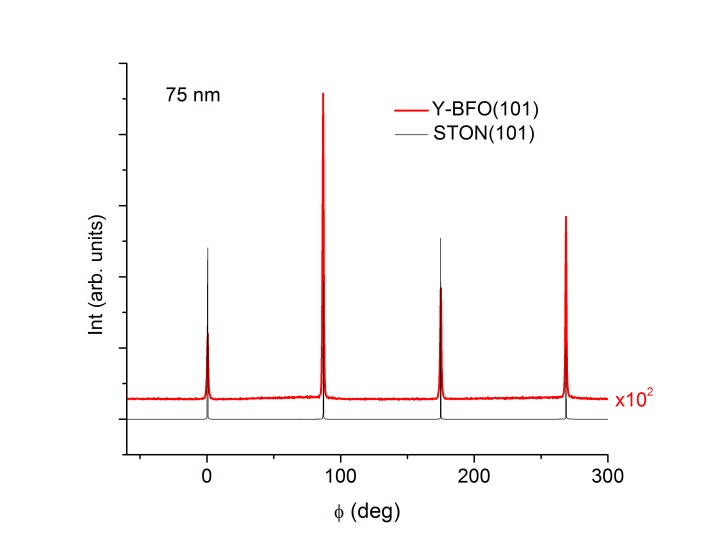*** |

Figure S1. X-ray Φ-scans around (101) plane of Y-BFO films and its corresponding STON substrate.

Table S1.

| **Samples**  **Tickness (nm)** | Thickness  errors (nm)^a^ | *a_out of plane_* (Å)^b^ | *a­_in plane_*  (Å)^c^ | *a*_out of plame_/*a*_in plane_ | L_┴_(nm)^d^ | ε_┴_ (%)^d^ | FWHM *_ω_*_(002)_  (deg) | L_II_ (nm)^e^ | α_tilt_ (deg)^e^ | Strain percent  (%)^b^ |
| --- | --- | --- | --- | --- | --- | --- | --- | --- | --- | --- |
| **22** | **22±1.06** | **4.0473±0.0066** | **3.910±0.0104** | **1.035** | **21±0.2** | **0.15±0.04** | **0.145** | **36±3.7** | **0.493±0.069** | **3.65±0.17** |
| 24 | 24**±0.68** | 4.0234±0.0075 | 3.909±0.0124 | 1.029 | 23±2.5 | 0.90**±0.14** | 0.207 | 26±3.0 | 0.683±0.103 | 3.03±0.19 |
| **31** | **31±0.57** | **4.0217±0.0055** | **3.923±0.0058** | **1.025** | **28±2.5** | **0.59±0.09** | **0.210** | **29±3.4** | **0.662±0.099** | **2.99±0.14** |
| 35 | 35±0.40 | 4.0218±0.0041 | 3.911±0.0039 | 1.028 | 50±1.6 | 0.41±0.02 | 0.354 | 38±3.9 | 0.677±0.062 | 2.99±0.10 |
| 46 | 46±0.41 | 4.0331±0.0009 | 3.926±0.0054 | 1.027 | 54+3.3 | 0.38±0.13 | 0.281 | 37±4.3 | 0.630±0.076 | 3.28±0.02 |
| 60 | 60±0.07 | 4.0163±0.0009 | 3.927±0.0069 | 1.023 | 59±12.0 | 0.47±0.04 | 0.693 | 39±2.7 | 0.884±0.043 | 2.85±0.02 |
| **61** | **61±0.37** | **3.9834±0.0041** | **3.954±0.0025** | **1.007** | **60+8.7** | **0.63±0.07** | **1.125** | **14±2.0** | **1.543±0.255** | **2.01±0.10** |
| **75** | **75±0.17** | **3.9726±0.0017** | **3.951±0.0047** | **1.005** | **69+11.0** | **0.45**±0.07 | **0.437** | **25±1.5** | **1.279±0.056** | **1.73±0.04** |

^a)^ the standard errors are provided by the software used for the evaluation of thickness via spectrometric ellipsometry measurements

^b)^ errors represent the standard error of the mean value calculated from the positions of the four (00*l*) reflections for each sample

^c)^  errors are extracted from the standard deviation of intercept of  linear regression of *d_hkl_*  values of asymmetric (*hkl*) reflections against (sinψ)^2^ plots, ψ being the tilted angle corresponding each asymmetric (*hkl*) plane used.

^d)^ errors are extracted from the standard deviation of intercept (for L_┴_) and the standard deviation of slope (for ε_┴_ (%)) of the  linear regression of W-H plots derived from the *θ-ω* scans of the symmetric (00*l*) reflections for each sample.

^e)^ errors are extracted from the standard deviation of intercept (for L_II_) and the standard deviation of the slope (for α_tilt_) from the  linear regression of W-H plots derived from the *ω*-scans around the same (00*l*) reflections for each sample.


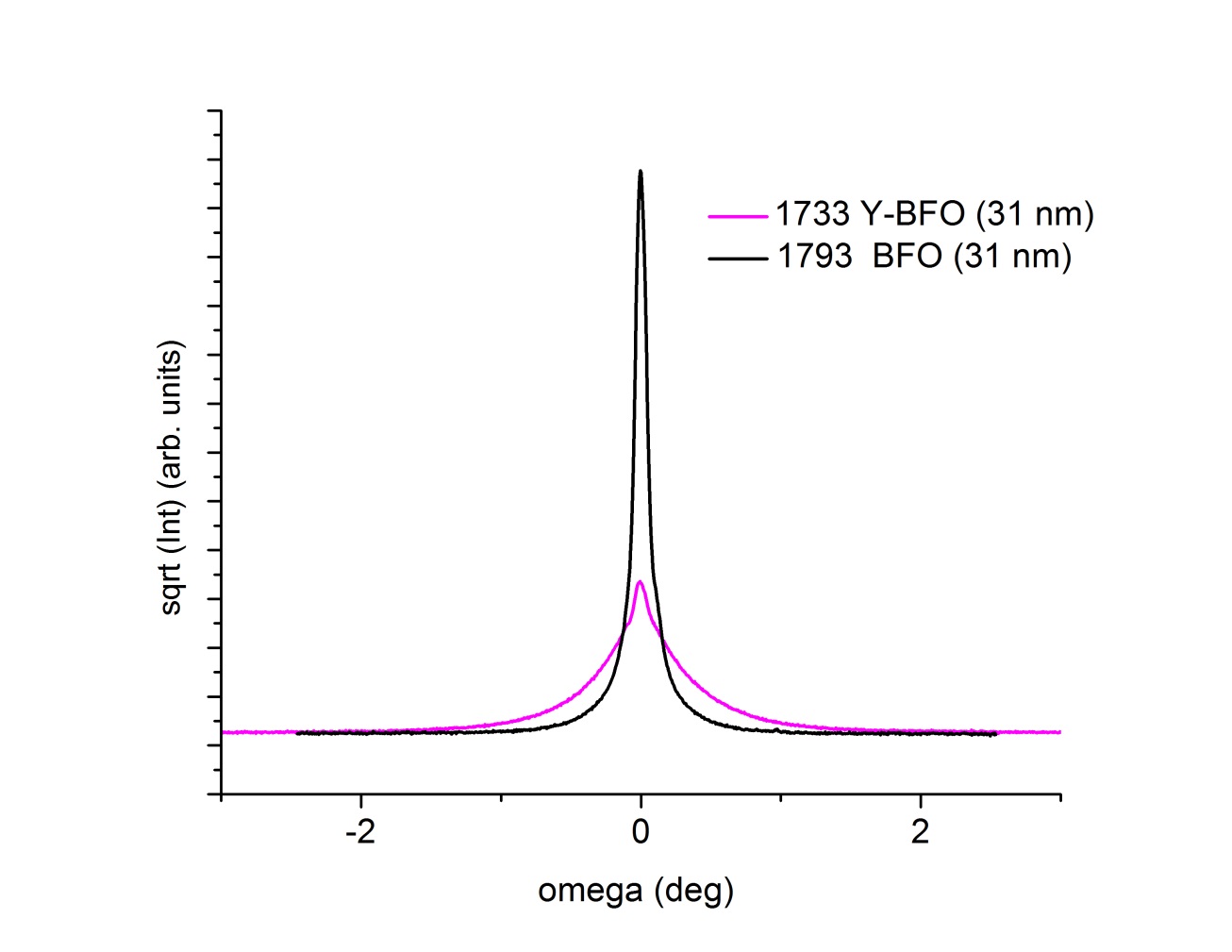


Figure S2. Comparison of superimposed XRD rocking curves of the Y-BFO(002) and BFO reflections from the films with the thickness of 31 nm. The intensities are in square root scale.

| 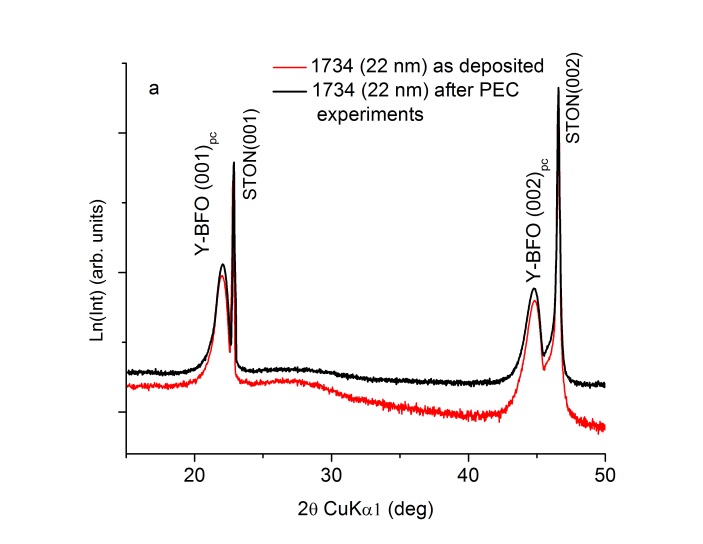 | 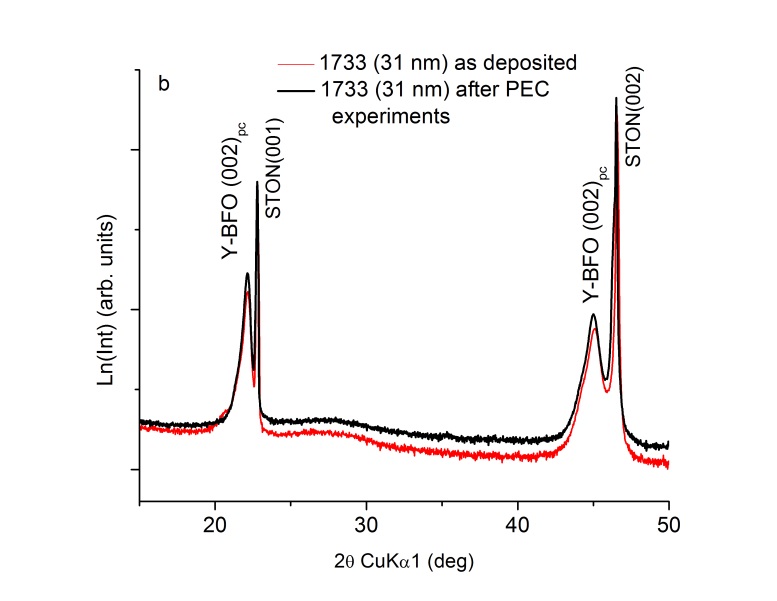 |
| --- | --- |
| 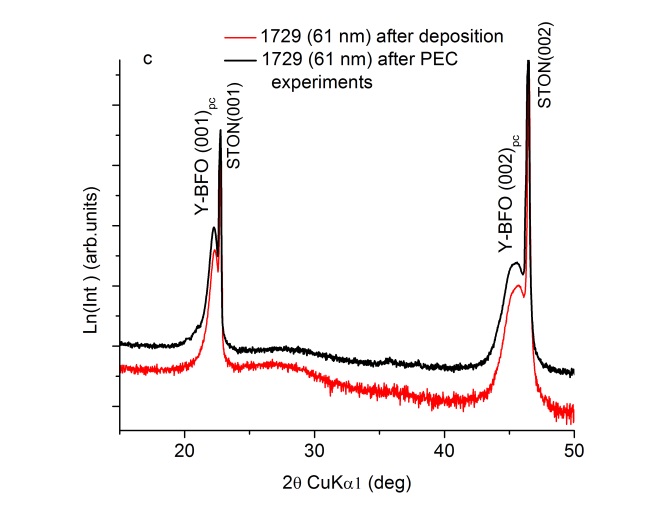 | 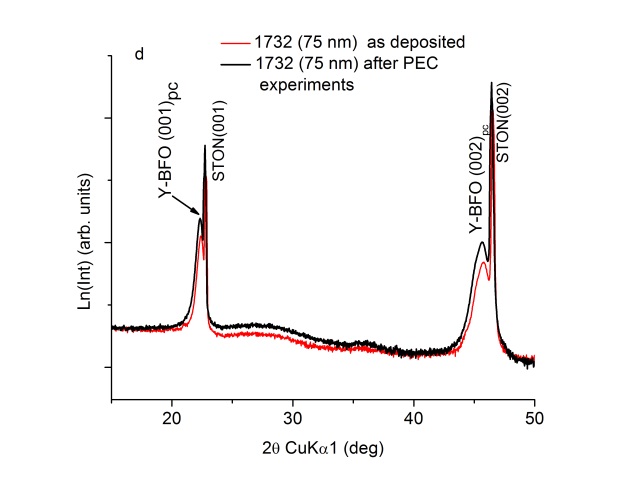 |

Figure S3. The XRD 2θ-ω scans of the Y-BFO thin films as deposited and after PEC experiments. The intensities are in logarithmic scale.

***Low magnification TEM images on Y-BFO films***

***
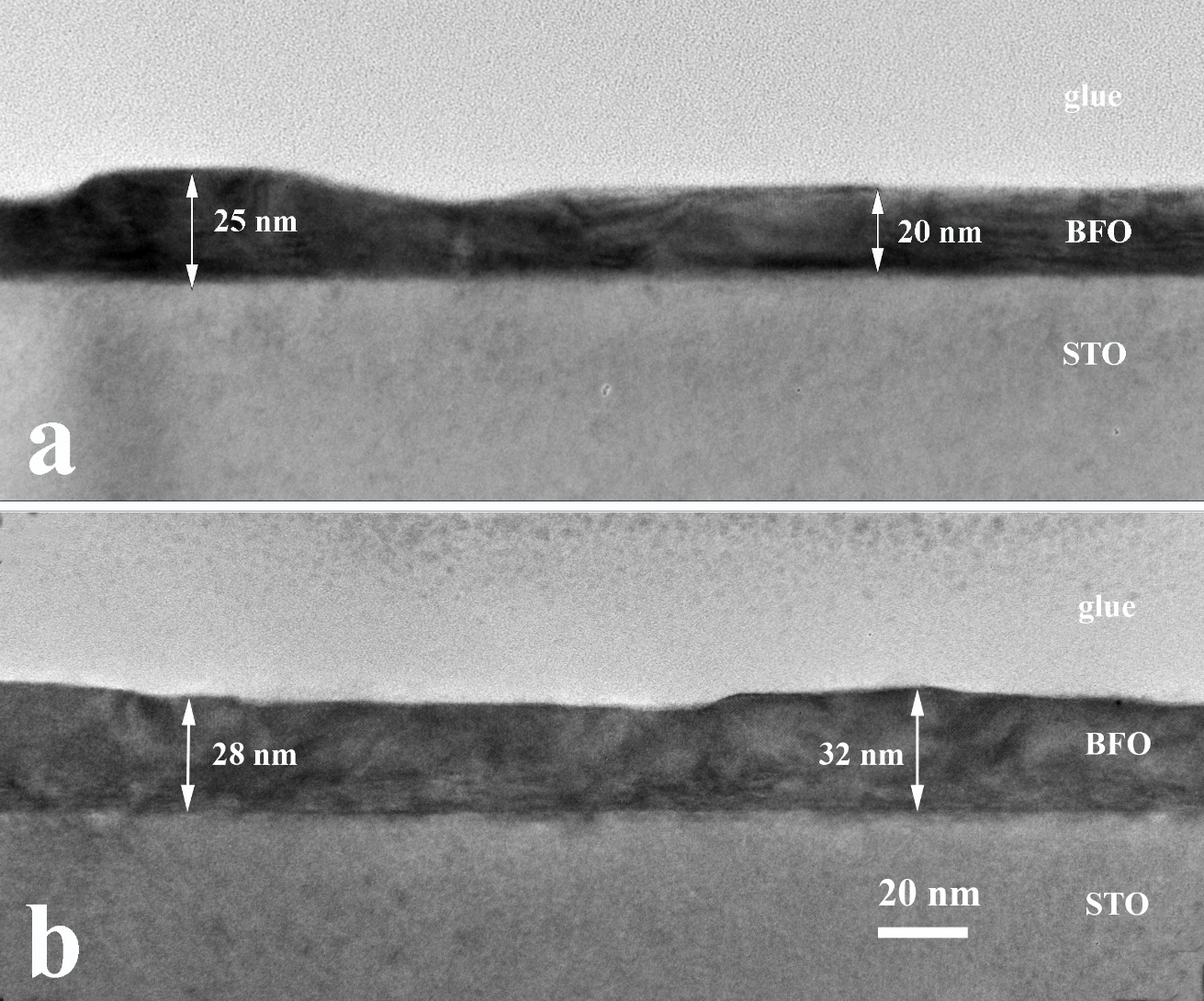
***

Figure S4. Low magnification XTEM image of the Y-BFO thin films

***PEC measurement on bare STON substrate***

***
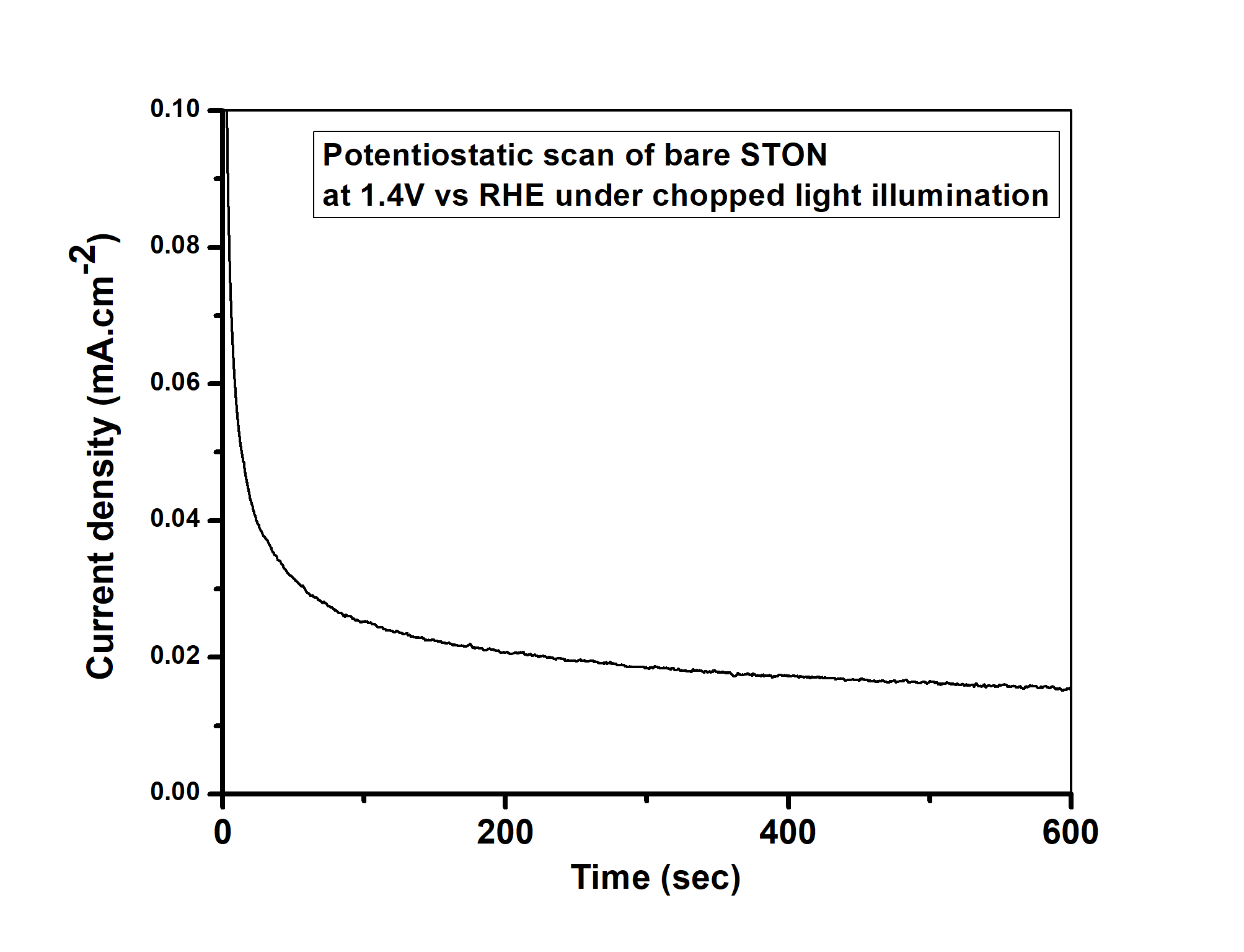
***

Figure S5. The potentiostatic scan at 1.4V vs RHE on bare STON substrate under chopped light illumination

***Optical properties of Y-BFO thin films and Nb:SrTiO3 used substrate.***

The optical properties of Y- BFO thin films and Nb:STO substrate were investigated by spectroscopic ellipsometry in the 250-1700 nm range of wavelength with a step of 2 nm at an 700 angle of light incidence.

The optical model used for the calculation of both the refractive index and the extinction coefficient values for Y-BFO sample considers a stack of 3 layers: the substrate, the Y-BFO thin film and the top rough layer. It was assumed that the top rough layer consists of equally distributed quantities of air and Y-BFO (50%-50%). The optical data of the Nb:STO substrate were calculated before the PLD and compared with the literature values [2]. Both the refractive index and the extinction coefficient values for the Y- BFO thin film were calculated by fitting the experimental data with a sum of tree Gauss oscillators. This model was described for the BFO thin film by C. Himcinschi et al [1]. Based on the generated values of the extinction coefficients for the Y-BFO thin film and the Nb:STO substrate, the absorptivity coefficient α values have been obtained using the relation:

*α=4πk/l*, where *k* is the extinction coefficient and *l* is the sample thickness value.

The absorptivity coefficient values plotted as a function of Y-BFO thin films thickness, at the irradiation wavelength value, are presented in Figure S6.


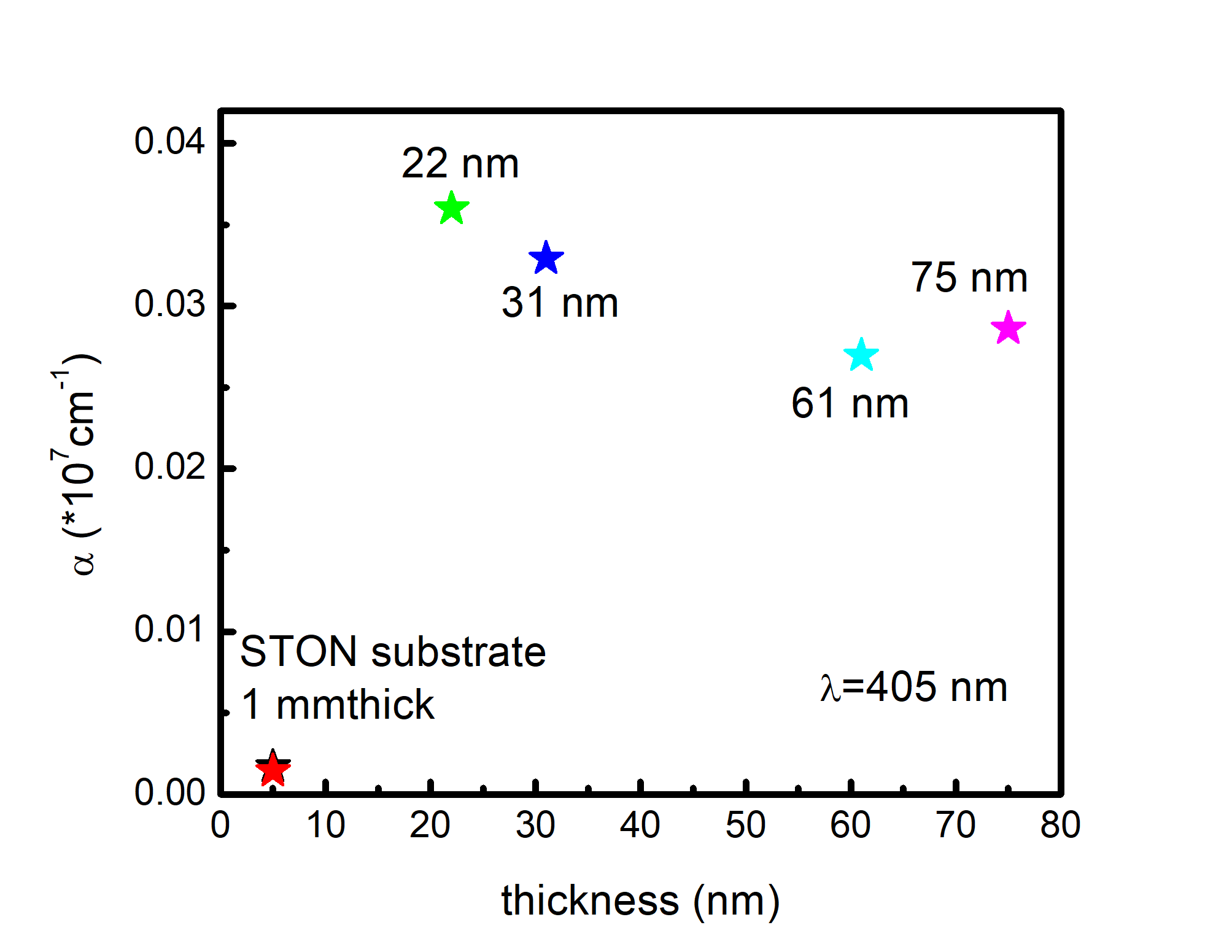


Figure S6. The absorptivity coefficient values for Y-BFO thin film and Nb:STO substrate at the irradiation wavelength obtained from ellipsometry measurements. The black star symbol correspond to the STON absorptivity value reported in Ref. 2.

***References***

***1.*** C. Himcinschi et al, Applied Physics Letters 106, 012908 (2015).

***2.*** Handbook of Optical Constants of Solids, pp 1042-1044, Palik E.D. (ed.), Academic Press, Orlando.
